# Comparison of three different lactic acid bacteria-fermented proteins on RAW 264.7 osteoclast and MC3T3-E1 osteoblast differentiation

**DOI:** 10.1038/s41598-023-49024-1

**Published:** 2023-12-07

**Authors:** Jae-Young Kim, Hyun Ji Song, Sejin Cheon, Seokyoung An, Chul Sang Lee, Sae Hun Kim

**Affiliations:** 1 College of Life Sciences and Biotechnology, Korea University, Seoul, 02841 Republic of Korea; 2 Institute of Life Science and Natural Resources, Korea University, Seoul, 02841 Republic of Korea

**Keywords:** Biotechnology, Microbiology

## Abstract

Osteoporosis is a state of bone weakening caused by an imbalance in osteoblast and osteoclast activity. In this study, the anti-osteoporotic effects of three proteins fermented by lactic acid bacteria (LAB) were assessed. Commercial proteins sodium caseinate (SC), whey protein isolate (WPI), and soy protein isolate (SPI) were fermented by LAB strains for 48 h. The fermented products (F-SC, F-WPI, and F-SPI, respectively) were used in an in vitro osteoclast and osteoblast-like cell model to assess their effects on bone health. Despite no difference in the results of TRAP staining of RANKL-induced osteoclastogenesis, F-WPI and F-SPI were effective in normalizing the altered gene expression of osteoclastogenesis markers such as TRAP, Nfatc1, RANK, and ATP6v0d. F-SPI was also effective in modulating osteoblasts by enhancing the expression of the osteoblastogenesis markers T1Col, Col2a, and OSX to levels higher than those in the SPI group, indicating that protein characteristics could be enhanced through bacterial fermentation. Moreover, these boosted effects of F-SPI may be involved with isoflavone-related metabolism during LAB-fermentation of SPI. These results demonstrate the potential of LAB-fermented proteins as dietary supplements to prevent bone loss. However, further understanding of its effects on balancing osteoblasts and osteoclasts and the underlying mechanisms is needed.

## Introduction

Osteoporosis is a major skeletal disease worldwide, particularly in aging societies^1^. Osteoporosis occurs due to the imbalanced action of osteoclasts and osteoblasts, which decreases skeletal stability through disruption of bone formation and resorption^1, 2^. Osteoblasts originate from mesenchymal stem cell precursors and regulate bone formation during remodeling or healing, whereas osteoclasts originate from hematopoietic progenitor cells^3^. Osteocytes, the principal mechanosensory cells of bone tissue, are terminally differentiated osteoblasts that maintain bone structure by contributing to the bone remodeling process by balancing the activities of osteoblasts and osteoclasts^4^. Among several subtypes of osteoporosis, primary osteoporosis is divided into two types including postmenopausal and senile osteoporosis, which is related with estrogen deficiency and aging^5^. Senile osteoporosis is a primary age-related bone metabolic disease characterized by bone loss and decreased bone fragility^6^. Calcium supplementation or bisphosphonates are the drugs of choice for clinical treatment of osteoporosis^7^. However, long-term treatment with these drugs can cause side effects such as esophagitis and osteonecrosis; therefore, new safe strategies are urgently needed^8^.

Sodium caseinate (SC), whey protein isolates (WPI), and soy protein isolates (SPI) are widely used protein sources in industrial research. SC is a variable multicomponent mixture containing casein monomers and is used as a source of protein in a wide range of processed food products^9^. Currently, the application of SC has been extended to encapsulation, stabilization, and generation of edible films, beyond their use as proteins^10^. However, as SC has been investigated primarily as a protein source, attempts to apply SC to osteoporosis have not been made. WPI is a milk protein obtained after the precipitation of casein during cheese production^11^. WPI is widely used as a food ingredient because of its high nutritional value, such as peptides, glycopeptides, and components promoting bone mineralization, which includes calcium, lactates, and phosphates^12^. In older women, WPI supplementation can block bone loss when a high bone turnover rate occurs during menopause^13^. SPI, which act as transporters of bioactive ingredients, are well recognized worldwide for its nutritional and functional value^14^. Several studies have suggested that soy protein consumption can lower blood lipid levels, decrease type 2 diabetes occurrence, and increase bone density^15^. Moreover, soy proteins inhibit density reduction in the femur bones of an osteoporotic rat model^16^. SPI is composed almost exclusively of two major proteins, namely β-conglycinin and glycinin^17^, which have been shown to have anti-osteoporotic effects in an ovariectomized animal model^15^. Emerging research has demonstrated better immune-boosting effects of fermented soy protein isolates (F-SPI) by functional bacteria than those of SPI alone^18^. Therefore, bacterial fermentation of protein sources may exert more beneficial effects than the source before fermentation.

Lactic acid bacteria (LAB) are lactic acid-producing gram-positive bacteria that are widely distributed in nature and are associated with various health benefits. For instance, some LABs possess antimicrobial effects against pathogenic microorganisms such as *Salmonella* and *Listeria*, and therefore are utilized in the food industry^19^. The most common health-promoting LABs are classified as probiotics which is defined as “live microorganisms that, when administered in adequate amounts, confer a health benefit on the host”^20^. Among various probiotics, the Lactobacillaceae family is the most well-known and used in the probiotic industry^21^. This includes strains such as *Lactobacillus acidophilus, Lacticaseibacillus rhamnosus, Lactobacillus gasseri*, and *Lactiplantibacillus platarum* that were formerly classified under *Lactobacillus*^22^. Recent studies suggest that LAB fermentation can improve protein applicability by modulating protein characteristics and functions. For example, *Lactobacillus plantarum* fermentation increases the protein and starch digestibility and viscosity in sorghum flour^23^. In addition, black soybean extracts fermented with *Lacticaseibacillus rhamnosus* GG and *Bifidobacterium animalis* subsp. *lactis* BB-12 showed greater hair growth-promoting effects than non-fermented black soybean extracts^24^.

Thus, the present study was conducted to screen for the best fermenting combination of LAB and three proteins (SC, WPI, and SPI) that exhibited the highest hydrolytic capacity. Furthermore, the anti-osteoporotic abilities of these LAB-fermented proteins were assessed using MC3T3-E1 mouse pre-osteoblasts and Receptor activator of nuclear factors κB ligand (RANKL)-induced osteoclastogenic RAW 264.7 mouse macrophage cells in vitro.

## Results

### Effects of LAB-fermented proteins on the proliferation of RAW 264.7 osteoclasts and MC3T3-E1 osteoblasts

**Figure 1:**
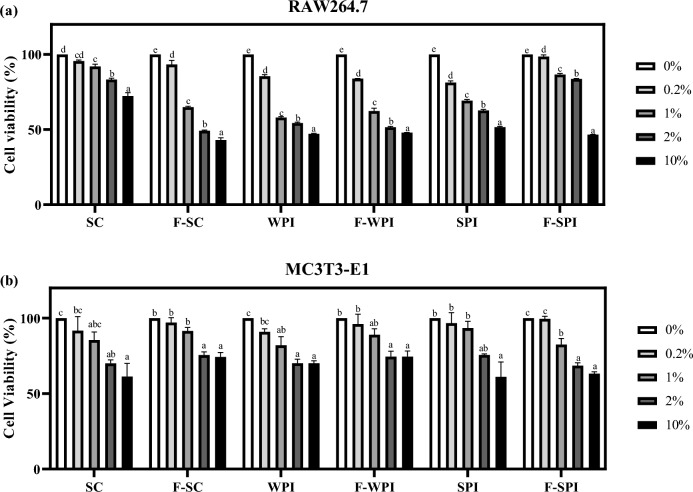
Cell viability of RAW 264.7 and MC3T3-E1 cells treated with LAB-fermented proteins for 48 h. (**a**) Cell viability of RAW264.7 cells pre-treated with SC, F-SC, WPI, F-WPI, SPI, F-SPI in various concentrations (0, 0.2, 1, 2, and 10%) for 48 h. (**b**) Cell viability of MC3T3-E1 cells pre-treated with SC, F-SC, WPI, F-WPI, SPI, and F-SPI in various concentrations (0, 0.2, 1, 2, and 10%) for 48 h. Results are expressed as means ± SE (*n* = 3). ^abcd^Means in the same series with different lowercase superscript letters are significantly different (*P* < 0.05).

To determine the optimal dosage for in vitro assays, the cell viability of RAW 264.7 and MC3T3-E1 cells treated with different dosages of LAB-fermented proteins were measured using the MTT assay (Fig. 1). Treatment of LAB-fermented proteins affected cell viability in a dose dependent manner. The results have shown that a 0.2% treatment of WPI, SPI, F-SC, and F-WPI maintained the viability of RAW 264.7 significantly higher than 80% (*P* < 0.05; Fig. 1a). Additionally, 1% treatment of SC adjusted RAW 264.7 cell viability to 92% (*P* < 0.05), and 2% treatment of F-SPI leveled RAW 264.7 cell viability to 83.7% (*P* < 0.05). For MC3T3-E1 cells, a 1% treatment of all protein samples maintained cell viability at levels higher than 70% (*P* < 0.05; Fig. 1b). According to the ISO 10993-5:2009, a reduction in cell viability higher than 30% is considered cytotoxic^25^. Therefore, these protein doses were considered tolerant in cellular application and used for further in vitro assays.

### Effects of LAB-fermented proteins on the differentiation of RAW 264.7 osteoclasts and MC3T3-E1 osteoblasts

**Figure 2:**
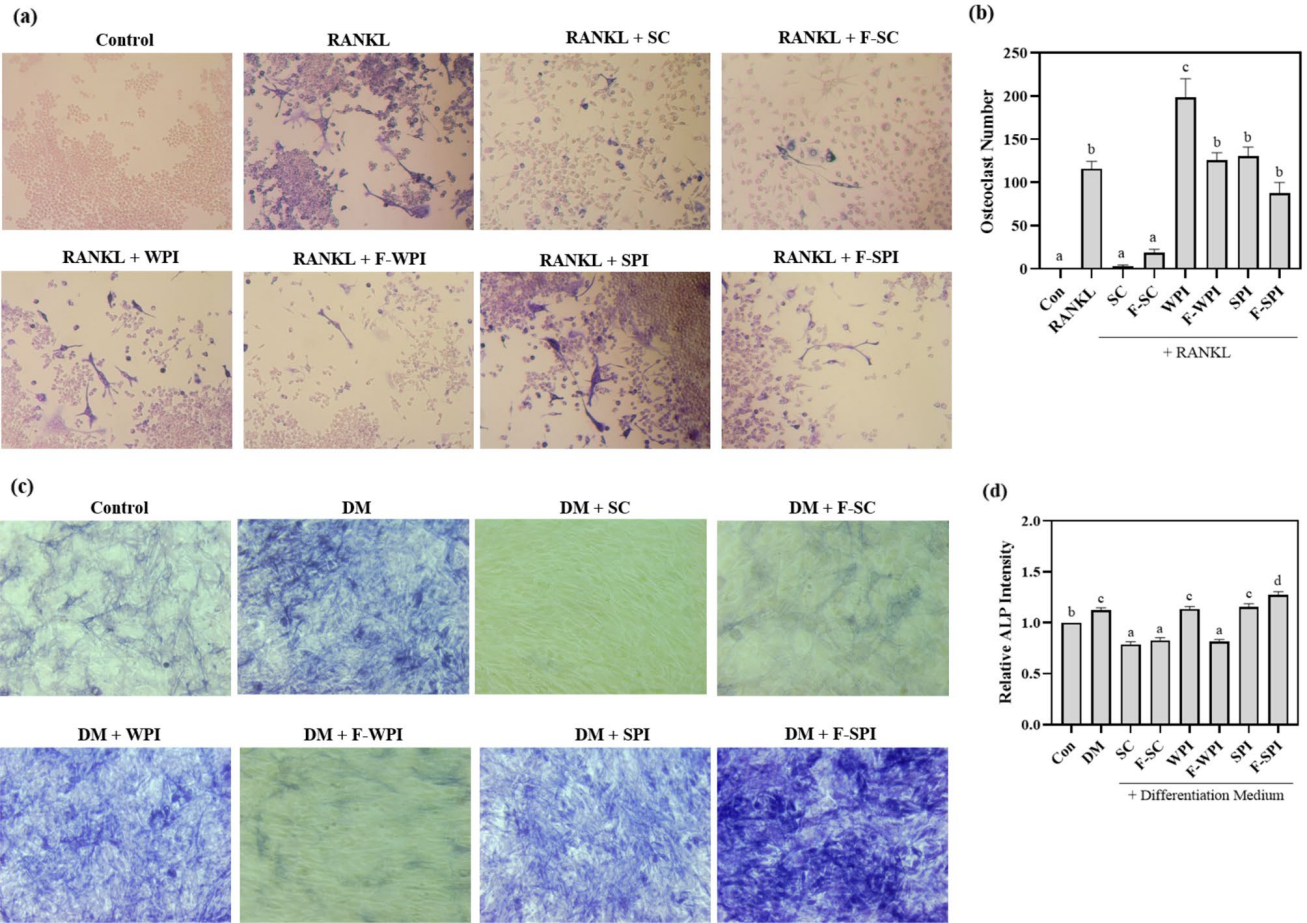
Effects of LAB-fermented proteins on differentiation of RAW264.7 and MC3T3-E1 cells. (**a**) TRAP staining of RANKL-induced RAW264.7 cells pre-treated with SC, F-SC, WPI, F-WPI, SPI, or F-SPI. (**b**) Number of mature osteoclasts in TRAP-stained RANKL-induced RAW264.7 cells pre-treated with SC, F-SC, WPI, F-WPI, SPI, or F-SPI. Results are expressed as means ± SE (*n* = 3). (**c**) ALP staining of differentiated MC3T3-E1 cells pre-treated with SC, F-SC, WPI, F-WPI, SPI, or F-SPI. (**d**) ALP staining intensity of MC3T3-E1 cells pre-treated with SC, F-SC, WPI, F-WPI, SPI, or F-SPI relative to the control group. Results are expressed as means ± SE (*n* = 3). ^abcd^Means in the same series with different lowercase superscript letters are significantly different (*P* < 0.05).

To determine the effects of LAB-fermented proteins on osteoclast differentiation, RANKL-induced RAW 264.7 cells were pre-treated with LAB-fermented proteins for 24 h. TRAP staining was done to confirm the formation of mature multinucleated osteoclasts. As a result, TRAP-positive multinucleated cells were observed in WPI and SPI treated cells (Fig. 2a). WPI and SPI, showed no effect on osteoclast differentiation reduction in both fermented and non-fermented types (Fig. 2b). Interestingly, treatment of SC and F-SC significantly reduced osteoclast differentiation induced by RANKL in RAW264.7 cells (Fig. 2b). To determine the effects of LAB-fermented proteins on osteoblast differentiation, MC3T3-E1 cells were pretreated with LAB-fermented proteins before differentiation induction. ALP staining was done to confirm the formation of osteoblasts. WPI and SPI maintained differentiation media-induced osteoblast formation, while SC, F-SC, F-WPI reduced differentiation significantly (*P* < 0.05; Fig. 2c,d). F-SPI was the only treatment effective in increasing osteoblast formation (*P* < 0.05; Fig. 2d). Interestingly, the intensity of F-SPI treated cells were higher than SPI treated cells, implying that LAB fermentation may have enhanced the promoting effects of osteoblast differentiation. Taken together, these observations show that LAB fermented proteins affect osteoclast and osteoblast differentiation.

### Effect of LAB-fermented proteins on gene expression levels of bone resorbing-related markers in RANKL stimulated RAW 264.7 osteoclasts

**Figure 3:**
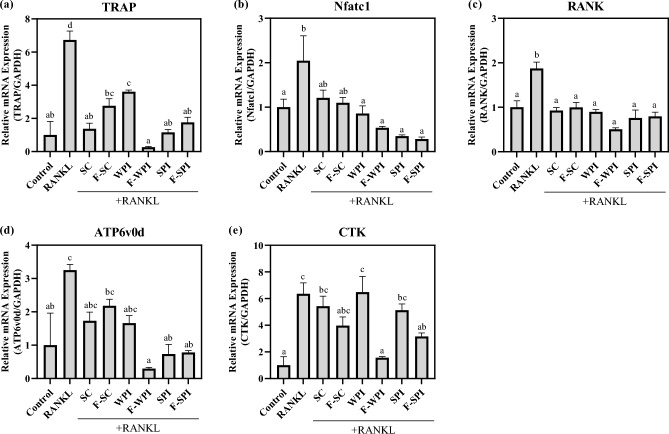
Effects of LAB-fermented proteins on gene expression levels of bone resorption-related markers in RANKL-induced RAW264.7 cells. Gene expression levels of (**a**) TRAP, (**b**) Nfatc1, (**c**) ATP6b0d, (**d**) RANK, and (**e**) CTK in RANKL-induced RAW264.7 cells pre-treated with SC, F-SC, WPI, F-WPI, SPI, and F-SPI. Results are expressed as means ± SE (*n* = 3). ^abcd^Means in the same series with different lowercase superscript letters are significantly different (*P* < 0.05).

The mRNA levels of genes related to bone resorption in samples treated with the LAB-fermented proteins were measured using RT-qPCR (Fig. 3). The mRNA expression levels of Tartrate-resistant acid phosphatase (TRAP), Nuclear factor of activated T-cells, cytoplasmic 1 (Nfatc1), ATPase, H + transporting, lysosomal 38 kDa, V0 subunit d2 (ATP6v0d), receptor activator of NF-κB (RANK) and Cathepsin K (CTK) were measured in RANKL-induced RAW264.7 osteoclasts (Fig. 3). RANKL injection has significantly upregulated the gene expression levels of all five bone resorption-related markers in RAW 264.7 cells compared to control (*P* < 0.05). The mRNA expression levels of TRAP were significantly downregulated by 3.0-fold in all three protein fermentations (*P* < 0.05) relative to the RANKL-stimulated group (Fig. 3a). Similar results were obtained for Nfatc1, ATP6v0d, and RANK (Fig. 3b–d). The expression levels of these genes significantly decreased by over 0.5-fold in all three protein treatment groups compared to those in the RANKL-stimulated group (*P* < 0.05). Interestingly, F-SPI significantly downregulated the increased gene expression levels of all five biomarkers to control levels (*P* < 0.05; Fig. 3). SPI reduced the RANKL-induced gene expression levels of all biomarkers, except CTK, to control levels (*P* < 0.05). Notably, RANKL-induced CTK expression was reduced by F-SPI and F-WPI treatments (*P* < 0.05; Fig. 3e). F-WPI suppressed the expression of all five biomarkers to levels similar to those in the control. SC and F-SC repressed the RANKL-induced gene expression of TRAP and RANK (*P* < 0.05). Taken together, these results suggested that F-WPI and F-SPI accelerated bone resorption by normalizing the gene expression levels of TRAP, Nfatc1, ATP6v0d, RANK, and CTK.

### Effect of LAB-fermented proteins on gene expression levels of bone forming-related markers in MC3T3-E1 osteoblasts

**Figure 4:**
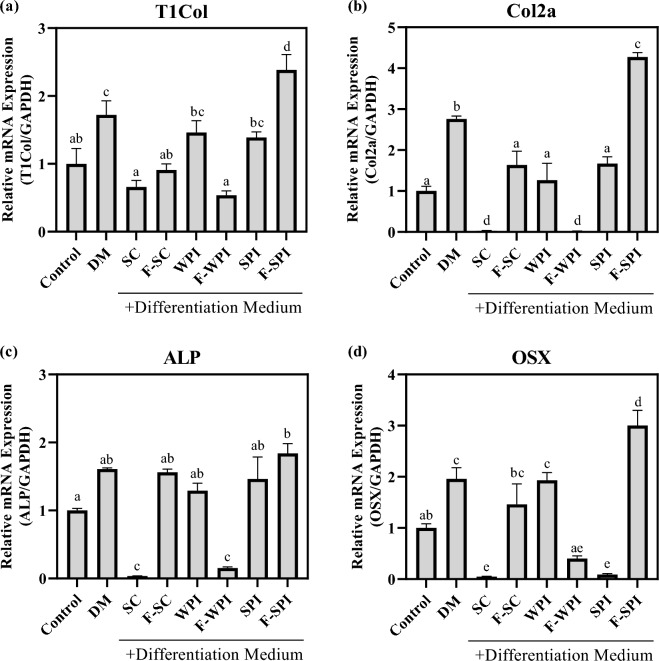
Effects of LAB-fermented proteins on gene expression levels of bone formation-related markers in MC3T3-E1 osteoblasts. Gene expression levels of (**a**) T1Col, (**b**) Col2a, (**c**) ALP, and (d) OSX in MC3T3-E1 osteoblasts pre-treated with SC, F-SC, WPI, F-WPI, SPI, and F-SPI. Results are expressed as means ± SE (*n* = 3). ^abcd^Means in the same series with different lowercase superscript letters are significantly different (*P* < 0.05).

The mRNA expression levels of bone forming-related genes in LAB-fermented proteins were measured using RT-qPCR (Fig. 4). Gene expression levels of type 1 collagen (T1Col), collagen type 2 alpha 1 (Col2a), alkaline phosphatase (ALP), and osteorix (OSX) were measured in MC3T3-E1 cells treated with differentiation medium (DM) containing glycerophosphate and ascorbic acid. F-SPI significantly upregulated T1Col, Col2a, and OSX by more than 0.6-fold compared to their expressions in the DM group (*P* < 0.05; Fig. 4a,b,d). Moreover, the F-SPI-induced ALP expression was similar to that observed in the DM group (Fig. 4c). This, along with the ALP staining results (Fig. 2c), confirmed that F-SPI is involved in the bone formation process. In contrast, F-WPI significantly downregulated the expression of all four biomarkers (*P* < 0.05) by more than 1.2-fold compared to that in the DM group, and ALP expression was significantly lower than in the control by 0.85-fold (*P* < 0.05). This difference was also observed during ALP staining (*P* < 0.05; Fig. 2c), indicating that F-WPI did not affect bone formation. SC, F-SC, WPI, and SPI did not enhance the gene expression of any of the four biomarkers, suggesting that their involvement in bone formation is scarce. These results indicated that F-SPI accelerated bone formation by modulating the gene expression levels of T1Col, Col2a, ALP, and OSX.

### Effects of fermentation on isoflavone compositions in SPI

**Figure 5:**
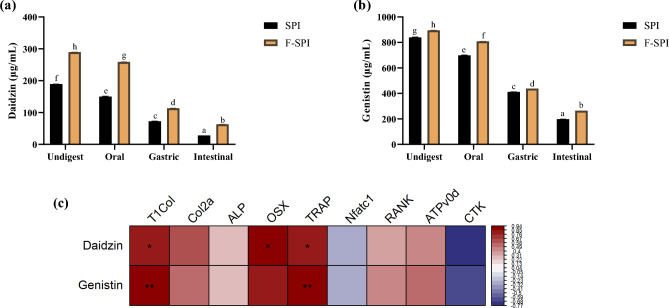
Concentration of SPI and F-SPI isoflavones before and after in gastrointestinal in vitro digestion. (**a**, **b**) Isoflavone levels of Daidzin, Genistin in SPI and F-SPI in vitro digests. Results are expressed as means ± SE (*n* = 3). ^abcdefgh^Means in the same series with different lowercase superscript letters are significantly different (*P* < 0.05). (**c**) Spearman correlation between gene expressions of MC3T3-E1, RAW264.7 cell markers, and isoflavone content in undigested SPI and F-SPI (*P < 0.05, ** P < 0.01, *** *P* < 0.001).

HPLC analysis revealed increased amounts of isoflavones such as daidzin and genistin in LAB-fermented SPI (*P* < 0.05; Fig. 5a,b). F-SPI was significantly higher in daidzin by 100.39 ± 0.20 μg/mL and genistin by 55.71 ± 3.1 μg/mL. In order to assess the bioavailabilty of daidzin and genistin in F-SPI, in vitro gastrointestinal digestion was conducted. Table 1 shows the changes to daidzin and genistin in the oral, gastric, and small intestinal compartments of SPI and F-SPI during digestion. Daidzin in SPI was reduced by 39.05 ± 1.14 μg/mL in the oral phase, 77.63 ± 1.07 μg/mL in the gastric phase, 44.48 ± 0.21 μg/mL in the intestinal phase; while daidzin in F-SPI was reduced by 30.76 ± 0.04 μg/mL in the oral phase, 145.48 ± 0.31 μg/mL in the gastric phase, 50.42 ± 0.35 μg/mL in the intestinal phase. Genistin in SPI was reduced by 140.7 ± 5.39 μg/mL in the oral phase, 287.42 ± 2.34 μg/mL in the gastric phase, 213.55 ± 0.76 μg/mL in the intestinal phase; while Genistin in F-SPI was reduced by 87.2 ± 0.68 μg/mL in the oral phase, 370.48 ± 0.69 μg/mL in the gastric phase, 173.14 ± 0.57 μg/mL in the intestinal phase. For further confirmation on whether bioactive components in F-SPI are isoflavones, spearman correlation analysis between gene expression levels of biomarkers in RAW264.7 osteoclasts, MC3T3-E1 osteoblasts and isoflavone levels were carried out (Fig. 5c). Correlation of bone formation-related marker T1Col, and bone resorption-related marker TRAP was proportional to daidzin and genistin level (*P* < 0.05). Bone formation-related OSX was proportional only to genistin level (*P* < 0.05). These results indicate that daidzin and genistin may be involved in the protective effects of F-SPI in RAW264.7 osteoclasts and MC3T3-E1 osteoblasts (*P* < 0.05).

**Table 1:** Isoflavone content in the undigest, oral, gastric, and small intestinal compartments of SPI and F-SPI during digestion.

Isoflavone (μg/mL)	SPI				F-SPI			
	Undigest	Oral	Gastric	Intestinal	Undigest	Oral	Gastric	Intestinal
Daidzin	189.35 ± 0.25f.	150.3 ± 1.08^e^	72.67 ± 0.36^c^	28.18 ± 0.15^a^	289.74 ± 0.09^ h^	258.98 ± 0.05^ g^	113.5 ± 0.26^d^	63.08 ± 0.144^b^
Genistin	839.87 ± 3.46^ g^	699.17 ± 2.08^e^	411.75 ± 0.89^c^	198.2 ± 0.16^a^	895.58 ± 0.5^ h^	808.38 ± 0.3f.	437.9 ± 0.38^d^	264.76 ± 0.19^b^

Results are expressed as mean ± SE (*n* = 3). ^abcdefgh^Means in the same series with different lowercase superscript letters are significantly different (*P* < 0.05).

## Discussion

Fermentation is the process of controlled microbial growth and enzymatic conversion of food components^26^. Probiotics such as *Lactobacillus* can participate in fermentation and produce beneficial compounds such as organic acids, bacteriocins, and peptides^27^. Furthermore, probiotic fermentation can improve digestion and absorption of insoluble macromolecular food components by converting them into smaller soluble substances^28^. Recent studies have suggested that probiotics can hydrolyze proteins through fermentation and produce metabolites such as free amino acids, isoflavones, and bioactive peptides, which exert beneficial effects on conditions such as senescence, neurodegenerative diseases, and inflammatory bowel disease^28, 29^. Therefore, this study aimed to determine the effects of LAB-fermented proteins on osteoporosis, a major symptom of senescence. Commercial proteins SC, WPI, and SPI as well as probiotics such as *Limosilactobacillus* were used for fermentation. In vitro assessments of osteoporosis were conducted using RAW264.7, an osteoclast-related model, and MC3T3-E1, an osteoblast-related model. The optimal treatment dosages for protein samples were determined using the MTT assay. Based on ISO cell toxicity guidelines, the highest concentration that did not reduce cell viability to levels lower than 80% was selected for further treatment^25^.

Bone homeostasis is an equilibrium state regulated by three key bone cells–osteoclasts, osteoblasts, and osteocytes–to maintain bone mass and integrity^30^. Osteoclasts are multinucleated cells formed near the bone surface that degrade the bone tissue^31^, whereas osteoblasts are bone-building cells that synthesize and deposit organic bone matrix proteins that mineralize the skeleton^32^. Osteocytes regulate the local mineral deposition in the bone matrix of mature skeletal tissue^33^. The activity of these major bone cells is affected by common risk factors such as senescence, postmenopause, and diet, which trigger imbalanced bone homeostasis along with symptoms such as osteoporosis and osteopenia^34^. Therefore, we assessed the ability of LAB-fermented proteins to restore uneven bone homeostasis in RANKL-induced osteoclasts and differentiated osteoblasts. RANKL is a protein ligand secreted by osteoblasts that induces osteoclast differentiation by binding to its receptor RANK^35^. To prevent osteoclast overexpression, osteoblasts secrete osteoprotegerin (OPG), which inhibits osteoclastogenesis by binding to RANK, thus preventing the action of RANKL^36^. The regulatory system of osteoclastogenesis is known as the RANKL/RANK/OPG pathway^35^. RANKL-induced osteoclastogenesis increases the number of mature osteoclasts that secrete bone-degrading acids and enzymes, such as TRAP^31^. Previous studies have shown that *Lactobacillus* strains exert osteoprotective effects by reducing osteoclastogenesis, as assessed by TRAP staining^30, 33^. Theses immunomodulatory effects trigger production of anti-osteoclastogenic inflammatory cytokines (IL-10) in T regulatory cells and repress the production of RANKL and osteoclastogenic inflammatory cytokines (IL-4, TNF-α) in T helper 17 cells^37^. Recently, treatment with LAB-fermented whey protein showed immunomodulatory effects by reducing the expression of IL-4 and TNF-α in an ulcerative colitis mouse model, demonstrating the potential of LAB-fermented proteins as novel immune-regulating therapies^38^. Moreover, the immune-related protective effects were greater in the LAB-fermented whey group than in the non-fermented whey group. Immune regulation is also known to reduce RANKL expression, leading to suppression of NFATc1^39^. NFATc1 activates the expression of genes that typify the osteoclast lineage such as CTK, ATP6v0d2, and TRAP^39, 40^. Our results showed that LAB-fermented proteins have osteoclastogenesis-modulating effects related to RANKL and NFATc1 signaling, and these effects were greater when using fermented proteins such as F-WPI and F-SPI. Despite no difference in TRAP-positive osteoclast numbers, TRAP expression was reduced by treatment of all protein sources, which implies that these effects may be because of osteoclastogenesis modulation through immunoregulatory mechanisms rather than regulation of osteoclast proliferation. However, further studies are needed to determine whether these protective effects can be attributed to immunoregulatory mechanisms.

Osteoblasts are bone-building cells that originate from mesenchymal stem cells (BMSCs)^41^. BMSCs differentiate into osteoblasts in three main stages: cell proliferation, matrix maturation, and matrix mineralization^42^. Osteoblast lineage progenitor cells differentiate into pre-osteoblasts via cell proliferation, and these pre-osteoblasts differentiate into osteoblasts via matrix maturation^37^. Osteoblasts can also differentiate into osteocytes by matrix mineralization^37, 42^. When osteogenic differentiation is triggered, BMSCs form Col2a-coated surfaces, which enhance calcium deposition and RUNX2 expressions^43^. Overexpression of RUNX2 and high calcium deposits are known to induce differentiation of BMSCs into pre-osteoblasts^43^. Pre-osteoblasts and osteoblasts secrete ALP to provide a high phosphate concentration at the osteoblast cell surface during bone differentiation and synthesize T1Col to facilitate cell attachment and utilize ALP for bone mineralization^44, 45^. Moreover, the binding of osteoblasts or BMSCs to T1Col accelerates osteogenic differentiation by triggering pathways such as ERK1/2 signaling^43^. OSX activates the transcription of osteoblastogenic markers during the maturation of pre-osteoblasts to osteoblasts, and once matured, osteoblasts produce OCN to regulate bone quality by aligning biological bone minerals parallel to the collagen fibrils^46^. In this study, F-SPI was the only protein to have significantly increased osteoblastogenesis marker T1Col, Col2a, and OSX expressions. F-SPI was more effective than SPI in normalizing osteoclastogenesis and improving osteoblastogenesis, proving that the bone-related functions of SPI can be enhanced by LAB-mediated fermentation. These findings are consistent with previous research; for instance, isoflavone-rich dry fermented soybean product supplementation for 15 weeks in senescence-accelerated mice increased bone mineral density and relative bone length^47^. In addition, mice fed with isoflavone-rich bacterial fermented soybean diet for four weeks showed reduced calcium deposition in the arterial wall, which is known to occur when osteoporosis occurs, and increased bone mineral density^48^. Soybeans are rich in isoflavones, which possess oxidation-, inflammation-, and osteoporosis-related effects. Among various types of isoflavones, daidzin and genistin mixtures reduced osteoclastogenic activities in RANKL-induced RAW264.7 cells^49^. Moreover, genistin-rich extracts enhanced osteoblastic differentiation in MC3T3-E1 cells^50^. Therefore, in this study isoflavone profiles of daidzin and genistin before and after fermentation were compared to identify potential bioactive compounds contributing to the greater bone-protective effects in F-SPI than SPI. As a result, amounts of daidzin and genistin were significantly increased in F-SPI, and theses isoflavones were positively correlated with in vitro gene expressions of T1Col, OSX, and TRAP. Daidzin and genistin are major soy isoflavones that exert weak estrogenic effects, which may prevent postmenopausal bone loss^51^. Moreover, the concentrations of daidzin and genistin were higher in F-SPI than SPI throughout all phases of in vitro digestion. This implies that potential bioactive components in F-SPI, daidzin and genistin, may survive digestion and exhibit systemic effects when orally consumed. These observations align with previous studies, suggesting that isoflavones are bioavailable even after digestion^52, 53^. Interestingly, daidzin is bioconvertable to equol by gut microbial metabolism^54^. Equol is a secondary metabolite of daidzin known for its high estrogenic and antioxidative activity, which helps prevent osteoporosis^55^. Thus, when orally consumed, the interactions of the gut microbiota and the daidzin in F-SPI may induce secondary metabolite productions, which may subsequently contribute to the efficacy of F-SPI. Therefore, further in vivo studies are needed to fully determine the effects of F-SPI in a gut-bone interactive setting. Despite numerous reports on the effectiveness of SPI on bone health, studies comparing the functions and compositional profiles of fermented and non-fermented materials are scarce, and therefore this research is rare in its attempt to do so. Not only can LAB metabolize isoflavones, but they can also degrade amino acids into oligopeptides using cell-envelope proteinase and peptidases^56^. The bioactive peptides produced in turn may possess calcium binding abilities, which have positive effects on bone homeostasis^57, 58^. The fermented-protein treatments used in this study were all byproducts of protein hydrolysis that increased amounts of amino acids such as tryptone, leucine, and serine (Supplementary Fig. S1). Interestingly, although all three candidates were protein sources, F-SPI was the only booster of osteoblastic activity. SPI is a vegetable protein source with no lactose and dietary fibers, making it easier to digest than other plant-sourced proteins. WPI is also an easily digested protein containing low levels of fat, lactose and high levels of cysteine. However, SC exists in a micelle form that slows down the process of full digestion^59^. This could have affected bioavailability of LAB metabolic precursors in each protein source, and subsequently produce fermentation end products with different efficacies. In our study, treatment with SC normalized T1Col gene expression in an osteoblastogenesis-induced environment but inhibited Col2a, ALP, and OSX expression to levels lower than the normal state. F-SC treatment also normalized T1Col and Col2a expression but inhibited other osteoblastogenesis-related markers to levels lower than those in the normal group. Moreover, SC and F-SC treatments did not normalize the expression of osteoclastogenesis markers such as ATP6v0d and CTK. The 1% treatment of SC and F-SCs did not affect cell viability, which suggests that SC and F-SC have inhibitory effects on osteoblastic activity, and are not suitable candidates for preventing bone loss. Notably, despite both being easily digestible proteins, F-SPI and F-WPI exhibited different impacts on in vitro models. This could be because LABs obtain distinct protease profiles depending on bacterial strain type—F-WPI was made by a *Pediococcus pentosaceus* strain, while F-SPI was made by a *Limosilactobacillus fermentum* strain^60^. WPI fermentation increased effects on normalizing gene expression levels of osteoclastogenic markers. However, both WPI and F-WPI failed to heighten DM-induced osteoblastic activity. These results align with those from a previous clinical study in which 184 elderly individuals were given a whey protein nutritional supplement for 13 weeks but showed little effect on improving bone mineral density^61^.

Based on the results of this study, F-SPI was identified as a potent candidate, exhibiting bone-protective effects in an in vitro model. However, this study had some limitations that warrant discussion. First, only in vitro experiments were conducted to examine the health benefits of F-SPI. Osteoblastogenic and osteoclastogenic activities of F-SPI were significant in in vitro osteoblast and osteoclast models. However, these results cannot represent the bioavailability of F-SPI bioactive compounds, and the possible interactions between the intestine and bone when consumed as food supplements. In order to determine bioavailability of bioactive compounds, in vitro digestion of F-SPI was conducted. Gel electrophoresis results showed that high molecular proteins observed in undigested samples were no longer intact after completion of the intestinal phase, which verifies that LAB-fermented protein digestion was conducted properly (Supplementary Fig. S2). In align with our findings, in vitro digestion of fermented soy beans increase accumulation of small molecular weight size fragments of free amino acids such as serine^62^. Moreover, the daidzin and genistin in F-SPI digests were higher than SPI digests in all phases of gastrointestinal in vitro digestion. These results suggest that oral consumption of F-SPI may provide higher amounts of isoflavones than SPI even after digestion. Therefore, these in vitro results need confirmation on the systemic effects of F-SPI in an in vivo study capable of observing the gut-bone interactions. Second, to confirm whether daidzin and genistin are the major metabolites responsible for the fermentation-induced features of F-SPI, the effects of F-SPI and the equivalent amount of these isoflavones should be compared in the same experimental models used in this study. Lastly, metabolites other than daidzin and genistin may contribute to the fermentation-induced characteristics of F-SPI. Spearman correlations only revealed significant relations among isoflavones, osteoblast T1Col, OSX and osteoclast TRAP gene expressions. Other markers assessed such as Col2a, ALP, Nfatc1, RANK, ATPv0d, and CTK showed no correlations with the isoflavones measured in this study. These results suggest possible activities of other bioactive compounds. Therefore, additional studies on the effects of F-SPI oral supplementation in an animal model and on F-SPI compositional analysis are in progress.

## Conclusion

This study aimed to evaluate the effects of LAB-fermented proteins in an in vitro osteoporosis model. Among the evaluated LAB-fermented proteins, F-WPI and F-SPI normalized osteoclastogenesis. Moreover, F-SPI enhanced osteoblastogenesis to levels greater than SPI and osteoblastic differentiation-induction media, suggesting the synergistic effects of probiotics and commercial foods through fermentation produced metabolites. Notably, osteoclastogenic gene expression results and TRAP staining in RANKL-induced RAW264.7 suggest the possibility that LAB-fermented proteins affect osteoclast differentiation through pathways other than RANKL/RANK signaling, such as regulation of inflammatory responses. Furthermore, LAB-fermentation of SPI induced modulations in isoflavone concentrations such as daidzin and genistin, which may have contributed to the effects of F-SPI in bone models. Therefore, in vivo studies are currently underway to determine the effects of LAB-fermented proteins in the presence of intestinal and skeletal interactions.

## Materials and methods

### Materials

Dairy- and plant-derived protein powder samples were purchased from commercial sources. The bacterial strains *Lactiplantibacillus plantarum* K15 (99% identity; accession no. NR_115605.1), *Pediococcus pentosaceus* SKP314 (98% identity; accession no. KX886792.1), and *Limosilactobacillus fermentum* 606 (98% identity; accession no. NR_113335.1) were obtained from the Food Microbiology Laboratory of Korea University (Seoul, South Korea). 16 s rRNA sequencing was conducted (Macrogen, Seoul, South Korea) for strain identification. All strains were grown in MRS broth (Kisan Bio, Seoul, South Korea) at 37 °C for 18 h. All strains were sub-cultured three times prior to use.

### LAB-fermented protein preparation

Bacterial fermentation of SC, WPI, and SPI were carried out.

SC powder was mixed with distilled water (1:20, w/v) and glucose (1:50, w/v). The mixture was sterilized by heating at 85 °C for 5 min. After cooling, the samples were streaked onto tryptic soy agar plates (Kisan Bio, Seoul, South Korea) to confirm sterilization. Thereafter, 1 × 10^10^ CFU/mL of *Lactiplantibacillus plantarum* K15 was added to each protein sample and fermented at 37 °C for 48 h. Samples were prepared at fermentation time points of 0 and 48 h for further analyses.

WPI powder was mixed with distilled water (1:20, w/v) and glucose (1:200, w/v). The mixture was sterilized by heating to 85 °C for 5 min. After cooling, the samples were streaked onto tryptic soy agar plates (Kisan Bio, Seoul, South Korea) to confirm sterilization. Thereafter, 1 × 10^10^ CFU/mL of *Pediococcus pentosaceus* SKP314 was added and fermented at 37 °C for 48 h. Samples were prepared at fermentation time points of 0 and 48 h for further analyses.

SPI powder was mixed with distilled water (1:20, w/v) and glucose (1:50, w/v). The pH was adjusted to 9.0 in order to increase SPI solubility. The mixture was sterilized by heating to 90 °C for 20 min. After cooling, the samples were streaked onto tryptic soy agar plates (Kisan Bio, Seoul, South Korea) to confirm sterilization. Thereafter, 1 × 10^10^ CFU/ml of *Limosilactobacillus fermentum* 606 was added and fermented at 37 °C for 48 h. Samples were prepared at fermentation time points of 0 and 48 h for further analyses.

### Determination of free amino acid residues

To measure the degree of proteolysis, the total amount of free primary amino acids was determined using an o-phthalaldehyde (OPA) spectrophotometric assay. OPA reagent was prepared by dissolving 40 mg of OPA in 1 mL methanol, to which 25 mL of 0.1 M sodium tetraborate, 2.5 mL of 20% (w/w) sodium dodecyl sulfate, and 100 μL β-mercaptoethanol were added. Distilled water was added to achieve a final volume of 50 mL. The pH values of each fermented protein product were measured at 0 and 48 h time periods and subsequently adjusted to the lower pH value among the two. In order to remove protein precipitates, SC and SPI fermented products were centrifuged (10,000*g*, 10 min, 4 °C) and filtered using 0.2 μm syringe filters (Sartorius, Sungnam, South Korea). WPI fermented product was centrifuged (8,000 g, 10 min, 4 °C) using Vivaspin (MWC 10,000) and filtered using 0.2 μm syringe filters (Sartorius, Sungnam, South Korea). Thereafter, 10 μL of each filtered samples were mixed with 180 μL of OPA reagent and incubated at room temperature (20 °C) for 2 min. The absorbance of each sample was measured at 340 nm using an Epoch microplate spectrophotometer (BioTek). Calibration curves were drawn using the amino acids obtained via protein digestion. Tryptone, leucine, and serine were used as the standards for SC, WPI, and SPI, respectively, at concentrations of 0–10 mM.

### Cell culture

RAW 264.7 murine macrophages were obtained from the Korean Cell Line Bank (KCTC, Seoul, South Korea). Cells were maintained in cell culture dishes containing Dulbecco’s modified Eagle’s medium (DMEM; Gibco, Dublin, Ireland) with 10% fetal bovine serum (FBS; Hyclone, MA, USA) and 1% penicillin/streptomycin (P/S; GE Healthcare, Chicago, IL, USA). The cells were then incubated in a humidified atmosphere (37 °C, 5% CO_2_).

MC3T3-E1 murine pre-osteoblasts were obtained from the American Type Culture Collection (ATCC, Manassas, VA, USA). The cells were maintained in cell culture dishes containing Minimum Essential Medium Eagle alpha modification (α-MEM, Gibco, Dublin, Ireland) with 10% FBS and 1% P/S. The cells were then incubated in a humidified atmosphere (37 °C, 5% CO_2_).

### Cell cytotoxicity assessment by MTT assay

An MTT (3-(4,5-dimethylthiazol-2-yl)-2,5-diphenyl tetrazolium bromide) assay was performed to assess the toxicity of fermented protein products on RAW 264.7 and MC3T3-E1 cells. The cells were seeded at a density of 2 × 10^4^ cells/well in 24 well plates. After 24 h of incubation, the cells were pre-treated with protein ferments in a dose-dependent manner (i.e., 0.2, 1, 2, and 10%) and incubated for 48 h. The cells were then treated with MTT solution (5 mg/mL, Sigma Aldrich) and incubated for 4 h. After removing the medium, the plates were washed with PBS and treated with dimethyl sulfoxide (DMSO; Sigma Aldrich). The absorbance was measured at 540 nm using an Epoch microplate spectrophotometer (BioTek, VT, USA), and the relative percentage of proliferation was calculated.

### Osteoclast differentiation assessment by TRAP staining

RAW 264.7 cells were seeded at a density of 3 × 10^4^ cells/well in 12-well plates. After 24 h, cells were treated with fermented protein products and differentiation media. α-MEM (Gibco, Dublin, Ireland) supplemented with Recombinant Human sRANK Ligand (RANKL, 100 ng/mL, Peprotech, Seoul, South Korea), 1% P/S, and 10% FBS was used for differentiation. The fermented protein product and differentiation medium were changed every 2 days. After 5 days, the cells were fixed with 10% formaldehyde for 10 min and stained with a tartrate-resistant acid phosphatase (TRAP) staining kit according to the manufacturer’s instructions (Takara, Shiga, Japan). Stained cells were washed with distilled water and dried at room temperature. Multinucleated osteoclasts with three or more nuclei were defined as TRAP-positive cells.

### Osteoblast differentiation assessment by ALP staining

The MC3T3-E1 cells were seeded at a density of 10^5^ cells/well in 12-well plates. After 24 h, cells were treated with protein fermentation and DM. α-MEM (Gibco, Dublin, Ireland) supplemented with 10 mM β-glycerophosphate (Sigma Aldrich), 50 μL/mL ascorbic acid (Sigma Aldrich), 1% P/S, and 10% FBS was used for differentiation. The fermented protein product and differentiation medium were changed every 2 days. After 6 days, the cells were stained using an alkaline phosphatase (ALP) staining kit according to the manufacturer’s instructions (Takara, Shiga, Japan). Cells were washed with PBS and observed using the Olympus CKX41 inverted phase contrast microscope (Olympus, Tokyo, Japan). ALP intensity was measured using Image J software (National Institutes of Health, MA, USA).

### Measurement of osteoclast and osteoblast differentiation related gene expression levels by reverse transcription quantitative real-time PCR (RT-qPCR)

RAW 264.7 cells were seeded at a density of 3 × 10^4^ cells/well in 12-well plates. After 24 h, the cells were treated with the fermented protein product and differentiation medium for 4 days. Both the fermented product and differentiation medium were changed every two days. Total mRNA was extracted using the TRIzol reagent (Life Technologies, Carlsbad, CA, USA) according to the manufacturer’s instructions. The concentration and purity of the extracted RNA were assessed using a NanoDrop spectrophotometer (BioTek, Winooski, VT, USA) and standardized to a final concentration of 0.1 μg/μL. The cDNA was synthesized using a reverse transcription kit (Thermo Fisher Scientific). The PCR cycling conditions were 25 °C for 10 min, 37 °C for 120 min, and 85 °C for 5 min. RT-qPCR was performed using the Bio-Rad CFX96 Real-Time PCR Detection System (Bio-Rad, Hercules, CA, USA). Targeted genes were quantified using MG2 x qPCR MasterMix (SYBR green) (MGmed, South Korea), and the RT-qPCR cycling conditions were initial denaturation cycle at 95 °C for 10 min followed by 40 cycles of amplification at 95 °C for 15 s, annealing at 55–65 °C for 30 s, and extension at 70 °C for 5 s. The mRNA expression level of each target gene was analyzed and normalized to that of the internal standard gene GAPDH, using Bio-Rad CFX Maestro (Bio-Rad Laboratories). The primer sequences used in this study are listed in Table 2.

**Table 2:** Primer sequences used for qRT-PCR analysis.

	Gene	Sequence	Ta (°C)
Osteoclast differentiation genes	TRAP	F: 5′- ACT TCC CCA GCC CTT ACT ACC G -3′	57
		R: 5′- TCA GCA CAT AGC CCA CAC CG -3′	
	Nfatc1	F: 5′- TGC TCC TCC TCC TGC TGC TC -3′	58
		R: 5′- CGT CTT CCA CCT CCA CGT CG -3′	
	ATP6v0d2	F: 5′- ATC CAG GTC ACA CAT TCC AGC A -3′	58
		R: 5′- CGA CAG CGT CAA ACA AAG GCT TGT A-3′	
	RANK	F: 5′- AAA CCT TGG ACC AAC TGC AC -3′	53
		R: 5′- ACC ATC TTC TCC TCC CGA GT -3′	
	CTK	F: 5′- AGG CGG CTA TAT GAC CAC TG -3′	59
		R: 5′- CCG AGC CAA GAG AGC ATA TC -3′	
Osteoblast differentiation genes	Type 1 collagen	F: 5′-GTG AGA CAG GCG AAC AAG -3′	55
		R: 5′-CAG GAG AAC CAG GAG GAC -3′	
	Col2a1	F: 5′- AGG GCA ACA GCA GGT TCA CAT AC -3′	63.3
		R: 5′-TGT CCA CAC CAA ATT CCT GTT CA -3′	
	Osteocalcin	F: 5′-GCT TAA CCC TGC TTG TGA -3′	63.3
		R: 5′-TCC TAA ATA GTG ATA CCG TAG ATG -3′	
	OSX	F: 5′-CGC TTT GTG CCT TTG AAA T -3′	64.5
		R: 5′-CCG TCA ACG ACG TTA TGC -3′	
	ALP	F: 5′- CCA GCA GGT TTC TCT CTT GG-3′	59
		R: 5′-GGA ATG TTC CAT GGA GGT TG -3′	
Housekeeping gene	GAPDH	F: 5′-TCT CCT GCG ACT TCA ACA -3′	63.3
		R: 5′-CTG TAG CCG TAT TCA TTG TC -3′	

The MC3T3-E1 cells were seeded at a density of 10^5^ cells/well in 12-well plates. After 24 h, the cells were treated with the fermented protein product and differentiation medium for 5 days. Both the fermented product and differentiation medium were changed every 2 days. Total mRNA was extracted using the TRIzol reagent (Life Technologies, Carlsbad, CA, USA) according to the manufacturer’s instructions. The concentration and purity of the extracted RNA were assessed using a NanoDrop spectrophotometer (BioTek, Winooski, VT, USA) and standardized to a final concentration of 0.1 μg/μL. The cDNA was synthesized using a reverse transcription kit (Thermo Fisher Scientific). The PCR cycling conditions were 25 °C for 10 min, 37 °C for 120 min, 85 °C for 5 min. RT-qPCR was performed using the Bio-Rad CFX96 Real-Time PCR Detection System (Bio-Rad, Hercules, CA, USA). Targeted genes were quantified using MG2 x qPCR MasterMix (SYBR green) (MGmed, South Korea), and the RT-qPCR cycling conditions were an initial denaturation cycle at 95 °C for 10 min, followed by 40 cycles of amplification at 95 °C for 15 s, annealing at 55–65 °C for 30 s, and extension at 70 °C for 5 s. The mRNA expression level of each target gene was analyzed and normalized to that of the internal standard gene GAPDH using Bio-Rad CFX Maestro (Bio-Rad Laboratories, Hercules, CA, USA). The primer sequences used in this study are listed in Table 2.

### INFOGEST static in vitro simulation of gastrointestinal food digestion

The in vitro gastrointestinal INFOGEST protocol was carried out with slight modifications^63^. In brief, 10 g of freeze-dried SPI and F-SPI samples were mixed in 1 mL of simulated salivary fluid (pH 7, 37 °C) containing amylase (75 U/mL of digesta, Sigma Aldrich, MO, USA) for 2 min. Then, 2 mL of simulated gastric juice (pH 3, 37 °C containing pepsin (2000 U/mL of digesta, Sigma Aldrich) and lipase (60 U/mL, Sigma Aldrich) were added and incubated for 120 min. Subsequently, 4 mL of simulated intestinal juice (pH 7, 37 °C) containing pancreatin (100 U trypsin activity/mL of digesta) and bile (10 mmol/L of total digesta) were added and incubated for 120 min. The entire digestion process was conducted at 37 °C with continuous gentle mixing. Gastric digestion was stopped after 120 min by adjusting the pH to 7 with NaOH (1 mol/L), and the intestinal phase was stopped by putting the samples in an ice bath immediately. All samples were rapidly frozen in liquid nitrogen and stored for further analysis.

### Sample preparation for HPLC

Daidzin and genistin standards were purchased from Sigma-Aldrich. Each isoflavone standard was dissolved in 80% methanol. SPI and F-SPI samples were extracted by the method from Agilent Technologies (CA, USA) with slight modifications^64^. Briefly, 10 g of protein samples were combined with 50 mL of 100% methanol and 10 mL of aqueous 0.1 M HCl. Both samples underwent sonication for 20 min at room temperature (20 °C). Subsequently, the supernatant from each sample was separated and filtered using Whatman No. 1 filter paper. The solvent in each sample was removed using a rotary evaporator, and the final volume was adjusted to 10 ml of 80% methanol. Extracted SPI and F-SPI samples were filtered by a second filtration step using a 0.22 μm aqueous phase filter membrane (Sartorious, South Korea) and stored at 4 °C until analysis.

### HPLC instrumentation and chromatographic condition

Total isoflavone contents of SPI and F-SPI digests were determined using HPLC, per the following method^65^. Chromatography was performed on an Agilent 1260 II Liquid Chromatography system equipped with a G7111A quadruplex pump, G7129A autosampler, and G7114A variable wavelength scanning UV detector. Daidzin and genistin separation was performed on an Agilent Poroshell 120 EC-C18 chromatographic column (4.6 × 50 mm, 2.7 μm), with a detection wavelength of 260 nm, a mobile phase of 200 μL formic acid in 1 L distilled water (solvent A) and 100% acetonitrile (solvent B) at a flow rate of 0.8 mL/min with a gradient: 5–80% solvent B from 0 to 10 min, 80% from 10 to 12 min, and 80%-5% from 12 to 16 min at ambient temperature (30 °C), and an injection volume of 5 μL. Under these conditions, daidzin and genistin eluted at a retention time of 5.95 ± 0.003 min and 6.63 ± 0.004 min respectively.

### Statistical analysis

Statistical analyses were performed using IBM SPSS Statistics software (version 25.0; IBM, Armonk, NY, USA). One-way analysis of variance (ANOVA) with Tukey’s test was used to analyze the statistical differences between the mean values of the samples. Statistical significance was defined as *P* < 0.05. All figures were drawn using GraphPad Prism 9.0 (GraphPad Software, Boston, MA, USA). Correlation based analyses and visualization were performed using R studio (RStudio, United States) and related packages.

### Supplementary Information

Supplementary Information.

## Data Availability

The datasets generated during and/or analyzed during the current study are available in the GeneBank (NCBI-Nucleotide Database) repository as following and also summarized in Supplementary Table ST.1. (1) *Lactiplantibacillus plantarum* strain K15 16s ribosomal RNA gene, partial sequence under accession number NR_115605.1 (link: https://www.ncbi.nlm.nih.gov/nuccore/NR_115605.1) (2) *Pediococcus pentosaceus* strain SKP314 16s ribosomal RNA gene, partial sequence under accession number KX886792.1 (link: https://www.ncbi.nlm.nih.gov/nuccore/KX886792.1) (3) *Limosilactobacillus fermentum* strain 606 16s ribosomal RNA gene, partial sequence under accession number NR_113335.1 (link: https://www.ncbi.nlm.nih.gov/nuccore/NR_113335.1).
