# Supplementary material for: Comparison of three different lactic acid bacteria-fermented proteins on RAW 264.7 osteoclast and MC3T3-E1 osteoblast differentiation
Source: Sci Rep. 2023 Dec 7;13:21575. doi: 10.1038/s41598-023-49024-1 (PMC10703878; doi:10.1038/s41598-023-49024-1)
Supplement: Supplementary file 1 — Supplementary Information. [file 41598_2023_49024_MOESM1_ESM.pdf]

Comparison of Three Different Lactic Acid Bacteria-Fermented Proteins on RAW 264.7 Osteoclast and MC3T3-E1 Osteoblast Differentiation

Jae-Young Kim[1,2], Hyun Ji Song[1], Sejin Cheon[1], Seokyoung An[1], Chul Sang Lee[1,2], and Sae Hun Kim[1,2*]

[1] College of Life Sciences and Biotechnology, Korea University, Seoul 02841, Republic of Korea.

[2] Institute of Life Science and Natural Resources, Korea University, Seoul 02841, Republic of Korea.

Author ORCIDs: Jae-Young Kim (0000-0003-1937-9535), Hyun Ji Song (0009-0005-6629-7817), Sejin Cheon (0009-0007-0298-7450), Seokyoung An (0009-0008-8713-1121), Chul Sang Lee (0000-0001-5371-5366), Sae Hun Kim (0000-0002-0990-2268)

[*]Corresponding author

Prof. Sae Hun Kim, Ph.D.; Email address: saehkim@korea.ac.kr

Supplementary Figure S1. Total amounts of free primary amino acids through LAB-mediated proteolysis

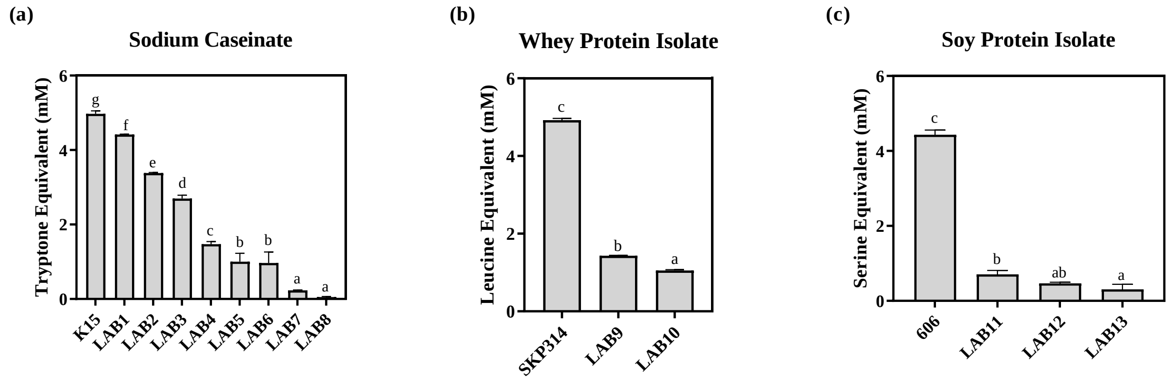

Effects of LAB fermentation on primary amino acid amounts of (a) SC, (b) WPI, or (c) SPI. Results are expressed as means ± SE (n = 3). [abcdefg]Means in the same series with different lowercase superscript letters are significantly different ($P < 0.05$).

Supplementary Figure S2. Sodium dodecyl-sulfate polyacrylamide gel electrophoresis of the undigested (U), supernatants (S) and pellets (P) of oral, gastric, intestinal phases from *in vitro* digestion of (a) SPI and (b) F-SPI

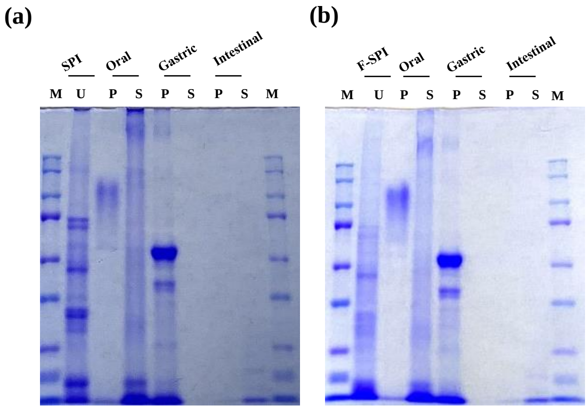

Supplementary Table ST.1 Identification of bacterial strains based on 16S rRNA gene sequencing data

Strain-ID	Source of isolation	Strain name/Genus species	16S rRNA gene (Number of nucleotides)	Accession number	Similarity of 16S rRNA gene sequence	
					Match/Total	Pct (%)
K15	*Kimchi*	*Lactiplantibacillus plantarum*	1493	NR_115605.1	1493/1494	99
SKP314	*Kimchi*	*Pediococcus pentosaceus*	1476	KX886792.1	1476/1506	98
606	*Kimchi*	*Limosilactobacillus fermentum*	1498	NR_113335.1	1498/1522	98
